# Reduction of Ultraviolet‐ and Heat‐Induced Aging Using Betulin‐Loaded Arginine–Caprylate Self‐Assembly: Randomized Double‐Blind Clinical Trials

**DOI:** 10.1111/srt.70361

**Published:** 2026-05-23

**Authors:** Koo Chul Kwon, Mi Jung Kim, Sang A Yoon

**Affiliations:** ^1^ Research Headquarters SEORIN COMPANY Co., Ltd. Seoul Republic of Korea

**Keywords:** anti‐inflammation, arginine‐caprylate, betulin, photoaging, ROS scavenging

## Abstract

**Background:**

Betulin exhibits potent antibacterial, anti‐inflammatory, and antioxidant properties, effectively preventing ultraviolet (UV)‐ and heat‐induced skin aging. Its limited solubility in cosmetic solvents restricts its application.

**Methods:**

To enhance betulin's applicability, a self‐assembled nanocarrier, arginine–caprylate ion pair loaded with betulin (B‐ACS), was developed. Structural properties, loading efficiency, skin permeability, and antioxidant activity were assessed. UVB‐induced damage was examined in human keratinocytes (HaCaT; *n* = 3) by measuring inflammatory cytokines and matrix metalloproteinases (MMPs) via ELISA. Three‐dimensional reconstructed human skin models were exposed to UVA (10 J/cm^2^) to evaluate collagen and aquaporin‐3 protection. A pilot randomized, double‐blind, placebo‐controlled clinical trial (*n* = 12, 2 weeks) assessed 3 % (w/w) B‐ACS cream on UV‐ and infrared‐induced skin damage.

**Results:**

B‐ACS showed a particle size of 236.9 ± 10.3 nm, 98.3 ± 1.3 % encapsulation efficacy, and superior reactive oxygen species (ROS) scavenging (IC50 = 4.87 ppm) compared to free betulin (IC50 = 8.26 ppm). In HaCaT cells, B‐ACS significantly reduced UVB‐induced inflammatory cytokine and MMP expressions (*p* < 0.05). In reconstructed skin, it prevented UVA‐induced collagen and aquaporin‐3 degradation. Clinically, 3% B‐ACS cream significantly improved elasticity (1.69‐fold), brightness (2.59‐fold), and pigmentation (1.91‐fold); transepidermal water loss (TEWL) and heat‐aging markers decreased 1.39‐fold and 1.63‐fold versus placebo, respectively (*p* < 0.05).

**Conclusion:**

B‐ACS enhances betulin's solubility and delivery, offering potent efficacy against UV‐ and heat‐induced aging.

## 1 Introduction

The skin, the largest organ in the body, serves as a protective barrier but is a potential target of oxidative stress caused by external damaging factors such as ultraviolet (UV) radiation, heat, ozone, and air pollutants, including various toxic chemicals. [1] Extrinsic aging accounts for up to 90% of visible skin changes commonly attributed to aging, including wrinkles, loss of elasticity, hyperpigmentation, and dryness. [2] Epidemiological data highlight the significant role of UV exposure in skin aging; for instance, up to 80% of visible aging in Caucasian women is caused by sun exposure, and globally, UV radiation contributes to 83% of melanoma cases, underscoring its link to photoaging and photocarcinogenesis. [3, 4] Oxidative stress plays a central role in both intrinsic and extrinsic aging, where reactive oxygen species (ROS) generated by stressors like UV and heat damage cellular components, including DNA, proteins, and lipids. This leads to inflammation, collagen degradation via matrix metalloproteinases (MMPs), and impaired barrier function, resulting in increased transepidermal water loss (TEWL). [5] Despite advances in antiaging strategies, research gaps persist, including the limited solubility and delivery of potent natural antioxidants and the need for formulations addressing combined UV‐heat stressors, which synergistically exacerbate ROS production and inflammation.

Human skin consists of three layers: the epidermis, dermis with appendages, and subcutaneous tissue. The dermis, rich in collagen and elastin, is particularly vulnerable to extrinsic stressors. UV radiation, comprising UVA (deeper penetration, chronic damage) and UVB (epidermal burns, DNA mutations), photoexcites skin molecules, triggering ROS formation and activating pathways like NF‐κB, leading to cytokine release and MMP upregulation. Heat, often from infrared radiation, similarly induces ROS and inflammatory infiltration, with combined UV‐heat exposure amplifying damage through enhanced MMP expression and tumor risk. [6] Exogenous antioxidants can mitigate these effects by scavenging ROS and reducing inflammation.

Phytochemicals, such as phenols, terpenoids, and phenolic acids, reduce UV‐mediated oxidative damage via antioxidant and anti‐inflammatory mechanisms. Among pentacyclic triterpenoids from birch bark, betulin stands out for its antimycotic, antimicrobial, antiviral, and antidiabetic properties. [7]

Previous cosmetic and clinical applications of betulin have primarily focused on its anti‐inflammatory effects in oleogels or its hydrating potential in nano‐spray formulations. However, these studies largely overlooked the synergistic damage caused by combined UV‐ and heat‐induced stressors, which exacerbate ROS production and collagen degradation more severely than either factor alone. [8, 9, 10, 11] This study advances the field by introducing a clinically validated nanocarrier specifically designed to address this complex aging pathway through integrated antioxidant and structural repair mechanisms.

To overcome betulin's inherent insolubility, conventional nanocarriers such as liposomes, nanoemulsions, and exosomes have been explored; however, they suffer from leakage and instability, lower encapsulation efficiency, and high production costs, respectively. [12, 13, 14] In contrast, arginine–caprylate self‐assembly (B‐ACS) represents a distinct class of surfactant‐free ion‐pair nanocarriers that forms spontaneously via electrostatic interactions between cationic arginine and anionic caprylate. This architecture provides superior long‐term stability through steric hindrance, achieves exceptionally high encapsulation efficacy (98.3 ± 1.3 %), and eliminates the need for synthetic surfactants, thereby reducing irritation risk and environmental footprint. Moreover, the arginine component uniquely enhances skin permeability via polyarginine motifs while serving as a nitric‐oxide precursor that promotes dermal collagen synthesis—synergies absent in conventional lipid‐based systems. [14, 15, 16, 17, 18, 19, 20, 21] Betulin was therefore incorporated into this arginine–caprylate platform to leverage the combined benefits of improved solubility, targeted delivery, and synergistic antioxidant activity.

Betulin was combined with arginine‐caprylate for self‐assembly, leveraging arginine's cationic nature for ion‐pairing and micelle formation, while providing synergy: arginine enhances skin permeability via polyarginine motifs, boosts collagen via nitric oxide, and amplifies betulin's ROS scavenging. [10, 22] Unlike other nanoformulations, this system is surfactant‐free, reducing irritation and carbon footprint, and offers superior stability and eco‐friendliness.

This study aimed to develop and evaluate betulin‐loaded arginine‐caprylate self‐assembly (B‐ACS) as a unique nanocarrier for enhanced betulin delivery and antiaging efficacy. Objectives included elucidating ion‐pair binding and self‐assembly, analyzing structural properties (e.g., size, zeta potential, morphology), assessing loading efficiency and skin permeability, evaluating antioxidant (DPPH scavenging) and anti‐inflammatory effects (cytokines, MMPs) in vitro, and verifying clinical efficacy via randomized trials on UV‐ and heat‐induced damage, measuring parameters like elasticity, brightness, pigmentation, TEWL, and carbonylated proteins.

## 2 Materials and Methods

### 2.1 Synthesis of B‐ACS

For the synthesis of B‐ACS, betulin (Sigma‐Aldrich, Inc., St. Louis, MO, USA; Cat. No. B9757) was dissolved in caprylic acid (Sigma‐Aldrich; Cat. No. C2875) by stirring at 60°C and 2000 rpm for 30 min. After dispersing arginine (Sigma‐Aldrich; Cat. No. A5006) in deionized Milli‐Q water at 0.57 M, the caprylic acid or betulin/caprylic acid mixture (0.57 M caprylic acid) was slowly added to the arginine dispersion and mixed with a homomixer (PRIMIX ROBOMIX, PRIMIX Corporation, Awaji, Hyogo, Japan) at 60°C and 2000 rpm for 10 min until a uniform liquid formed without any precipitates. Molar ratios were optimized from 10:1 to 1:10 (arginine:caprylate), with intermediate visual assessment for transparency indicating solubility enhancement, and the optimal 1:1 ratio selected based on stability. No further purification was performed. The macroscopic stability of B‐ACS was assessed at 50°C, 0°C, and 25°C for 4 weeks.

### 2.2 Structural Analysis

#### 2.2.1 1H ‐NMR

To validate the bonding states of arginine, caprylic acid, and betulin, 1H‐NMR analysis was performed. During the B‐ACS manufacturing process, deuterium oxide (99 atom% D) (Sigma‐Aldrich; Cat. No. 151882) replaced Milli‐Q water. For the structural analysis of caprylic acid and betulin, dimethyl sulfoxide‐d6 (99 atom% D; Sigma‐Aldrich; Cat. No. 156914) was used. 1H‐NMR spectra were recorded at 35.0°C using Avance III‐500 (NMR spectrometer; Bruker Instruments, Billerica, MA, USA) operating at 500 MHz in deuterium oxide solution. Tetramethylsilane was used as the internal standard.

#### 2.2.2 FT‐IR

The changes of the functional group were analyzed using Vertex 70 FTIR spectrometer with Hyperion 2000 microscope (Bruker Instruments) over a spectral range of 4000–500 cm^−1^ at a data acquisition rate of 1 cm^−1^ per point. The Opus 5.5 software (Bruker Instruments) was used for spectral development and deconvolution.

### 2.3 Thermal Analysis

A differential scanning calorimeter (DSC 1; Mettler‐Toledo, Columbus, OH, USA) was used to measure thermal properties. Endothermic heat flow and temperature were recorded with an accuracy of 0.0001 mW and 0.01 K, respectively. Measurements were conducted under a purified nitrogen atmosphere at a flow rate of 20 cc min^−1^ and heating rate of 10 K min^−1^.

### 2.4 Morphological Analysis

The morphology of B‐ACS was analyzed using a transmission electron microscope (TEM) (JEM‐2010 instrument; JEOL Ltd., Tokyo, Japan). Samples were stained with UranyLess EM Stain (22409; Electron Microscopy Sciences, PA, USA) on a 200‐mesh carbon grid. Size distribution and zeta potential were investigated using NanoSAQLA (Otsuka Electronics, Tokyo, Japan) and ELSZ‐Neo (Otsuka Electronics), respectively, and determined by dynamic light scattering with a particle size analyzer (Zetasizer Nano ZS; Malvern Instruments, Malvern, UK). Each experimental group was analyzed in at least 11 runs, performed in triplicate.

### 2.5 Betulin Determinations Using High Performance Liquid Chromatography (HPLC)

Betulin concentrations in the B‐ACS solution and receptor‐phase samples (transdermal delivery) were determined using HPLC. The HPLC (Agilent 1260 Infinity II; Agilent Technologies, Santa Clara, CA, USA) was conducted on a system with a Phenomenex Gemini RNX‐C18 (250 × 4.6 mm, 5 µm) column (Phenomenex; Cat. No. 00G‐4435‐E0), and samples were manually injected through an injector. Separation was achieved using a C18 column with a mobile phase at a flow rate of 1 mL/min, and betulin was detected at 254 nm. Methanolic betulin solutions were used as standards.

### 2.6 In Vitro Transdermal Release Experiments

The transdermal release experiments were conducted at 35°C using phosphate‐buffered saline (PBS; pH 7.4) as the receptor medium. Porcine back skin samples (2.5 × 2.5 cm^2^, 1 mm thick) were obtained from 6‐month‐old micropigs (body weight 20 ± 5 kg) through APURES Inc. (Gyeonggi, Republic of Korea), an approved commercial vendor for animal research materials. This study strictly adhered to the ARRIVE guidelines 2.0; notably, no live animals were utilized in any part of the experiments, as all tests were performed using *ex vivo* tissues (Franz Cell Membranes) procured under ethical standards compliant with animal welfare regulations. Further information regarding skin production and a completed ARRIVE checklist are provided in the Supplementary Information.

The manufactured FCMs were transported and stored at ‐20°C in a PBS‐impregnated state immediately after production, and were used within one week of production. The porcine skin sample was placed between the receptor and the Franz diffusion cell and then fixed to the transdermal device. In the Franz cell device, the stratum corneum faces the formulation‐applied compartment and the dermis faces the receptor compartment. The receptor was filled with PBS containing Tween 20 (0.5 %), which increased betulin solubility. ROUND LAB birch juice O/W cream (100 µL) was used as a vehicle, a cream containing 5 % (w/w) B‐ACS (total betulin contents in applied cream is 60 mg/kg) was used as an evaluation group, and a cream in which the same content of betulin was dispersed in the oil phase was used as a control group. The cream was applied to the stratum corneum of pig skin. To ensure the same viscosity, the same amount of B‐ACS or purified water was added to all creams. After 16 h, samples were collected and the amount of permeated betulin was quantified using HPLC. The experiments were performed in four independent replicates (*n* = 4), and the results are presented as mean ± standard deviation. [23]

### 2.7 DPPH Radical Scavenging Assay

The 2,2‐diphenyl‐1‐picrylhydrazyl (DPPH; 100 µM) radical was dissolved in 100% ethanol. The mixture was shaken vigorously and left to stand for 30 min at 23°C in the dark. The resulting dark‐blue DPPH radical solution was adjusted to an absorbance of 0.5 ± 0.05 at 517 nm. Test materials (arginine, caprylic acid, betulin, and B‐ACS at 0.1, 2, 5, and 10 ppm; 10 µL each) were added to 190 µL of this solution in a 96‐well microplate. The control consisted of 10 µL of 99% ethanol and 190 µL of the DPPH radical solution. A decrease in absorbance due to the addition of test compounds was measured at 734 nm using a microplate reader (Infinite 200 PRO; Tecan, Männedorf, Switzerland). The DPPH radical‐scavenging activity of the test compounds was expressed as a percentage of the remaining DPPH radicals at each time point. All samples were analyzed in triplicate. [24]

### 2.8 Cellular Study

#### 2.8.1 Cell Culture

HaCaT human keratinocytes (RRID: CVCL_0038) were used. Cells were derived from our existing laboratory stock (originally sourced from CLS/Cytion, Cat. No. 300493). Cells were authenticated by STR profiling and confirmed mycoplasma‐negative. The HaCaT cells were grown in Dulbecco's modified Eagle's medium supplemented with 10% fetal bovine serum (Biowest, USA) and 1% penicillin/streptomycin. Cells were incubated at 37°C in a humidified atmosphere containing 5 % CO_2_ and 95 % air. Subculturing was performed when the cells reached approximately 80 % – 90 % confluency.

#### 2.8.2 UV Irradiation and Cytotoxicity Assessment

Cytotoxicity was assessed using the water‐soluble tetrazolium salt (WST‐1) assay. HaCaT cells were cultured for 24 h before the addition of the WST‐1 assay solution (EZ‐CyTox; DoGenBio, Korea) at 10% of the medium volume, followed by incubation at 37°C for an additional 0.5 – 1 h. Absorbance was measured at 450 nm using a microplate reader (SpectraMax i3x Multi‐Mode Detection Platform; Molecular Devices, USA), with a reference absorbance recorded at 650 nm for correction. The results, expressed as the mean ± standard deviation from three independent experiments, were used to evaluate cell viability. The same method was applied to determine the UV stimulation conditions for assessing the anti‐inflammatory effects of B‐ACS. Cytotoxicity was measured in human keratinocytes exposed to UVA (0.25, 0.5, 0.75, 1.0 J/cm^2^ over 1.5 min) or UVB (12.5 25, 50, 75 mJ/cm^2^ over 1.5 min), and the measured values were compared with those of the negative control group to evaluate cell viability (data not shown). For subsequent evaluations, the UV irradiation doses were determined as UVA (0.75 J/cm^2^) and UVB (25 mJ/cm^2^). [25, 26, 27]

#### 2.8.3 Evaluation of the Protein Expression of Cytokines Using ELISA

HaCaT cells (1 × 10^5^) were incubated in 24‐well plates for 24 h. Cells were then treated with 1, 10, and 100 µg/mL of arginine, caprylic acid, betulin, and B‐ACS for 30 min, followed by UVB (25 mJ/cm^2^) irradiation, and further incubated for 24 h. The levels of CCL2/JE/MCP‐1, IL‐4, IL‐6, IL‐8/CXCL8, TNF‐*α*, MMP‐1, and MMP‐9 proteins were quantified using an ELISA kit for humans (R&D Systems, Minneapolis, MN, USA). Results are presented as the mean ± standard deviation of three independent experiments. [28]

#### 2.8.4 Evaluation of UVA‐Induced Photoaging Using Artificial Skin

A whole human skin tissue model (KeraSkin, Biosolution Co. Ltd., Seoul, Korea) was purchased and treated with 100 µg/mL of B‐ACS for 30 min, followed by UVA (0.75 J/cm^2^) irradiation and a 24‐h incubation period. After incubation, the tissues were fixed using 4% formaldehyde. The fixed tissues were embedded in paraffin blocks and sectioned at 10 µm for H&E and Masson's trichrome staining. For immunostaining, the sectioned tissues were treated with a 20,000‐fold diluted mouse anti‐collagen III antibody (Abcam, Cambridge, UK) or a 2,000‐fold diluted mouse anti‐aquaporin 3 antibody for 3 h at 25°C, followed by incubation with HRP‐conjugated secondary antibodies for 45 min at room temperature.

### 2.9 Clinical Study

#### 2.9.1 Clinical Study Overview

This randomized, double‐blind, placebo‐controlled clinical study was conducted from December 23, 2024, to February 12, 2025. The study protocol was reviewed and approved by the Institutional Review Board of the Korea Institute of Dermatological Sciences on December 20, 2024 (approval number: KIDS‐BDK097‐SNC) prior to participant recruitment. Written informed consent was obtained from all participants prior to their enrollment in the study. All procedures adhered to the ethical guidelines of the Declaration of Helsinki and the Bioethics and Safety Act of the Republic of Korea. As this was an exploratory pilot evaluation of cosmetic efficacy—not involving investigational medicinal products—clinical trial registration in a public registry was not required under current Korean cosmetic research regulations, and thus no registration number was obtained.

The study was intentionally designed as a pilot trial to provide preliminary evidence of the efficacy and feasibility of 3% (w/w) B‐ACS cream in an intra‐subject, split‐site format. A sample size of *n* = 12 was selected based on published cosmetic and dermatological pilot studies employing similar instrumental endpoints, such as elasticity and pigmentation. Because the primary objective was effect‐size estimation and proof‐of‐concept rather than definitive population‐level inference, a formal a priori power calculation was not performed; however, this cohort size was sufficient to detect statistically significant differences (*p* < 0.05).

Twelve adult women (aged 25–66 years; mean 45.58 ± 12.57) with Fitzpatrick skin types II–IV and ITA° values of 29°–55° participated voluntarily. The enrollment was limited to female participants to align with the primary target consumer group for the tested product and to reduce inter‐gender variability in skin physiology and responses to UV and heat stress. Participant flow is summarized in Figure S1.

The three application sites (B‐ACS cream, placebo cream, and untreated) were randomly assigned to the left upper arm using computer‐generated block randomization (block size 3) via R 3.5.3. Allocation concealment was maintained by an independent statistician using sealed opaque envelopes, which were opened only after enrollment and baseline measurements. To ensure rigorous double‐blinding, both participants and investigators remained blinded to the formulation identities; the B‐ACS and placebo creams were identical in appearance, texture, and odor, labeled only with codes. All 12 participants completed the 2‐week study without drop‐outs or protocol deviations, with each individual serving as her own control.

Skin irritation was induced by irradiating 76.75 mJ/cm^2^ of UVA + UVB light over 3 min at an interval of 2.5 cm on the left upper arm using a solar simulator (Multiport Simulator 601‐300 W; Solar Light Company, Inc., PA, USA). Subsequently, the left upper arm was randomly divided into three regions: test substance application, control substance application, and non‐application areas. Initial device measurements were performed before treatment. During the 2‐week test period, the evaluation areas were applied twice daily with equal amounts of either B‐ACS 3% containing cream or vehicle cream after washing in the morning and evening and allowing for full absorption, or nothing was applied. The placebo cream was the identical O/W cream base (same vehicle, viscosity, and all excipients) excluding the B‐ACS complex. This allowed us to evaluate the total clinical benefit of the betulin‐loaded self‐assembly system as a single functional intervention. The same skin measurements were performed after 2 weeks. Before evaluation, the measurement sites were washed with the same cleanser and allowed to rest for 30 min in a constant temperature and humidity environment (temperature: 22 ± 2°C, humidity: 50 ± 5%). During the test period, participants were instructed to avoid using sunscreen, functional cosmetics, and body moisturizers that could affect the results. Additional procedures, such as packs and massages, were also prohibited.

The test manager monitored the occurrence of skin abnormalities, such as erythema, edema, scaling, itching, stinging, burning, tightness, and prickling, at the test site. Any observed reactions were recorded by grading skin abnormalities. No adverse reactions were observed during the study.

### 2.10 Clinical Assessment

#### 2.10.1 Skin Brightness Evaluation Using ANTERA 3D

Skin brightness was assessed using the ANTERA 3D system (Miravex, Dublin, Ireland). The same trained assessor measured the designated test sites (where B‐ACS cream, a negative control cream, or no treatment was applied) on the left upper arm of all participants. To ensure measurement reproducibility, images captured before the application of the test substance were superimposed onto subsequent images to maintain consistent measurement sites. The captured images were analyzed using the ANTERA CS software to obtain the mean CIE L*, a*, and b* values. The L* value, which represents skin brightness, was used in the analysis. An increase in the L* value from baseline and a significant increase (*p* < .05) at the test site compared with the control and untreated sites were interpreted as improvements in skin brightness.

#### 2.10.2 Skin Elasticity Evaluation Using Ballistometer

Skin elasticity was evaluated using a Ballistometer (Ballistometer BLS780; Dia‐Stron Ltd., Andover, UK). The same examiner measured the designated test sites. Data were analyzed using the dedicated MApp software, focusing on the coefficient of restitution as an indicator of skin elasticity. A higher coefficient of restitution value after the application of the test material, along with a statistically significant increase (*p* < .05) at the test site compared with the control and untreated sites, was considered an improvement in skin elasticity.

#### 2.10.3 TEWL Using Tewameter TM300

TEWL was measured using the Tewameter TM300 (Courage+Khazaka Electronic GmbH, Cologne, Germany). The examiner applied the probe with consistent pressure to the designated test sites, measuring TEWL three consecutive times at each site until stable readings were obtained. The average value was used for the analysis. Data were processed using the MPA CTplus, Tewameter TM300's dedicated software, which measures changes in the density of evaporated water (unit: g/m^2^/h). A decrease in TEWL compared with baseline, along with a statistically significant reduction (*p* < .05) at the test site compared with the control and untreated sites, was considered an improvement in skin barrier function.

#### 2.10.4 Evaluation of Pigmentation Using Mexameter

Skin pigmentation was assessed using the Mexameter MX18 (Courage+Khazaka Electronic GmbH). Measurements were taken three consecutive times at the same pressure, and the average value was calculated for analysis. The melanin index, which represents the melanin content of the skin, was used as the primary parameter. A decrease in the melanin index compared with baseline, with a statistically significant reduction (p < 0.05) at the test site compared with the control and untreated sites, was considered an improvement in skin pigmentation.

#### 2.10.5 Evaluation of Heat‐Induced Skin Aging Markers (Carbonylated Proteins) in a Hot and Dry Environment

To induce skin keratinization under high‐temperature and low‐humidity conditions, a medium‐sized dehumidifier (NED‐050P; Nawooel, Korea) and an infrared radiator (IR300; Daekyoung Co., Kyungki, Korea) were operated in a separate closed space to maintain specific environmental conditions (temperature: 40.00 ± 2°C, humidity: 22.00 ± 2%). All participants exposed both forearms to this environment for 20 min. Prior to instrumental analysis, keratinized skin samples were collected from both forearms (test material site, control material site, and untreated site) using an 18‐mm transparent tape (Scotch transparent tape; 3 M Company, USA). The tape was applied at a constant pressure for 2 s, then removed in the same direction, repeating the stripping process 20 times. The first layer of collected keratin was discarded, and only 20 stripped layers were used in the experiment. Sampling was performed at two time points: before product application (after exposure to high‐temperature and low‐humidity conditions) and after 2 weeks of product use.

To analyze the increase in heat‐aging markers (carbonylated proteins) in the stratum corneum under high‐temperature, dry conditions, a fluorescence microscope (Fluorescence Imaging System, Thermo Fisher, USA) and an image analysis program (ImageJ, National Institutes of Health, USA) were used. Fluorescence images were captured with a 10× objective lens, and the green value was analyzed as an indicator of carbonylated protein levels. A decrease in the green value compared with baseline, along with a statistically significant decrease (*p* < 0.05) in the test area compared with the control and untreated areas, was interpreted as an improvement in heat‐induced skin aging markers. [29]

### 2.11 Statistics

Statistical analysis was performed using SPSS 17.0 for Windows program. The Shapiro–Wilk test assessed normality for measurement values before and after applying the test substance. If normality was satisfied, a paired *t*‐test was performed; otherwise, a Wilcoxon signed‐rank test was used. For measurement intervals with three or more time points, the normality test was followed by either a repeated measures analysis of variance (ANOVA) for parametric data or the Friedman test for nonparametric data. The Bonferroni main effect test was used for post‐hoc analysis in the parametric case. For nonparametric data, pairwise comparisons were conducted using the Wilcoxon signed‐rank test, with the significance level adjusted via the Bonferroni correction method before performing the post‐test. However, when evaluating measurement values before and after applying the test substance under special conditions (continuity, environment factors, cleaning, etc.), repeated measures ANOVA was performed without a normality test. When comparing test, control, application, and non‐application areas, the Shapiro–Wilk test was used to assess normality for measurement values before and after use if two evaluation time points were present. If normality was satisfied, an independent *t*‐test was performed; otherwise, the Mann–Whitney U test was used. For three or more evaluation time points, repeated measures ANOVA was conducted without a normality test.

## 3 Results

### 3.1 Preparation and Structural Analysis of B‐ACS

**FIGURE 1:**
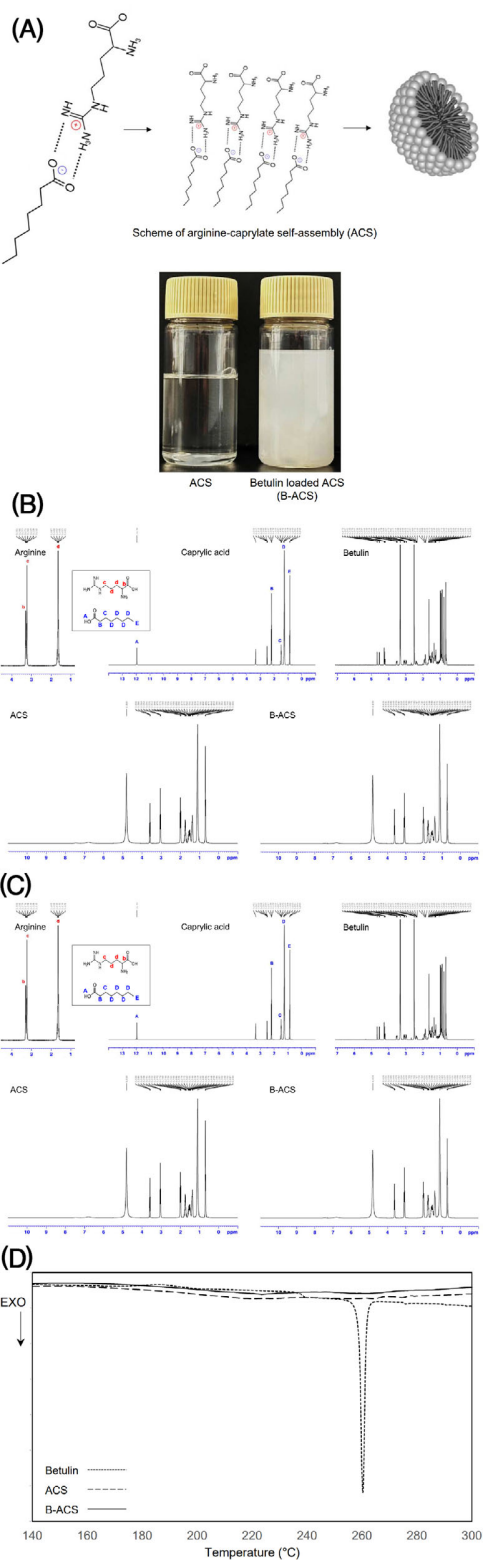
(**A**) ACS and B‐ACS formation scheme and images, (**B**) 1H‐NMR spectra, (**C**) FT‐IR spectra of arginine, caparylic acid, betulin, ACS, and B‐ACS, (**D**) DSC patterns of betulin, ACS, and B‐ACS.

To optimally prepare the arginine–caprylate ion pair, caprylic acid was stirred in aqueous arginine solutions at 60°C with molar ratios ranging from 10:1 to 1:10. The optimal molar ratio was determined based on two criteria: (1) increased solubility observed through excess arginine addition and (2) increased solubility of caprylic acid, which is insoluble in water. After the mixing reaction and subsequent resting at room temperature, the formation of an appropriate ion pair was confirmed by the appearance of a transparent aqueous solution, indicating increased solubility of arginine and caprylic acid. The optimal molar ratio was further confirmed as 1:1 by assessing macroscopic stability over 4 weeks at 50°C, 0°C, and room temperature. The arginine–caprylate ion pair self‐assembled due to the structural characteristics of arginine's polar portion and caprylate's nonpolar portion, forming micelles (Figure 1A). In addition, betulin, possessing a hydrophobic surface charge, can be supported on the micelle structure. When betulin dissolves in caprylic acid heated to 60°C, ion pair formation and subsequent self‐assembly occur in a similar manner, leading to the formation of arginine–caprylate self‐assembly (B‐ACS) containing betulin (Figure 1A).

The binding state of the arginine–caprylate ion pair was confirmed using 1H‐NMR analysis (Figure 1B). After ion pair formation, caprylate protons B–D showed an upfield shift in the ACS spectrum, with proton B, located closest to the carboxyl group, experiencing a pronounced shift. In the case of arginine, two major changes were observed: first, the upshift of proton C (near the guanidyl group) widened the peak gap with proton B (near the amine); second, proton D, appearing as a single peak, split into two. This peak splitting suggests that the rotational motion of the C‐C chain, to which proton D belongs, was reduced by intermolecular bonding among surrounding functional groups. In addition, the upfield shift of proton C near the guanidyl group peak suggests that the guanidyl group formed an ion pair with the carboxyl anion of caprylic acid, increasing electron density. The bonding states of the functional groups were identified by comparing the IR spectra of the single components with those of ACS (or B‐ACS), as shown in Figure 1C. The guanidyl peak of arginine at 1634 cm^−1^ showed a red shift after ACS (or B‐ACS) formation, suggesting that intramolecular bond lengthening occurred due to intermolecular bonding, thus identifying the location of hydrogen bond formation in arginine. In Figure 1C, the change in the 1705 cm^−1^ peak, signifying C = O, along with the −OH peak indicated by blue shading, confirmed that the carboxyl group of caprylate participated in bonding. The betulin peak in Figure 1B shows no change before and after B‐ACS formation, but the IR spectrum in Figure 1C shows the disappearance of the peak at the betulin terminal −CH_2_OH (2930 cm^−1^) after B‐ACS formation. These results suggest that betulin did not form bonds with other molecules but was loaded within ACS. Thermal analysis supported these findings, as shown in Figure 1D. No melting point peak was observed for betulin, a compound with a known melting point of 257°C, when contained within ACS. This confirmed that betulin, a poorly soluble component, was stable within ACS.

### 3.2 Morphology and Antioxidant Activity of B‐ACS

**FIGURE 2:**
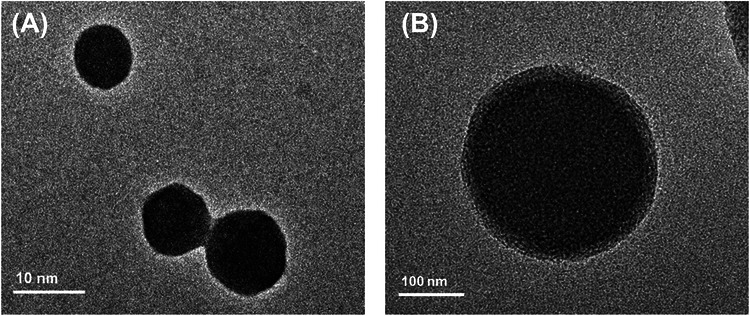
TEM images of ACS and B‐ACS.

To assess their morphology, ACS and B‐ACS were negatively stained and observed using a TEM at ×100k (Figure 2A) and ×40k (Figure 2B) magnification. Both structures appeared spherical, confirming their vesicular characteristics. Important factors to consider when designing nanocarriers for skin delivery include particle size, uniformity, and loading efficiency. The particle size, polydispersity index, and zeta potential of B‐ACS are listed in Table 1. The encapsulation efficacy of betulin was 98.3 ± 1.3%, and the ACS of 4.7 ± 0.1 nm was confirmed to swell up to a maximum level of 236.9 ± 10.3 nm depending on the loading of betulin. However, even after swelling, the polydispersity index of B‐ACS was 0.257, indicating a homogeneous vesicle population, and the zeta potential value (∼ ‐9.9 mV) did not change significantly before and after betulin loading. Although this low zeta potential may suggest potential instability, aggregation is prevented by steric hindrance from the ion‐pair structure and hydrophobic interactions, as evidenced by long‐term stability over 4 weeks. In vitro skin penetration experiments were performed by applying equivalent amounts of betulin dissolved in oil or B‐ACS to the O/W cream (viscosity: 10,000 cP; pH: 6.5, suitable for skin neutrality and spreadability) on porcine back skin (Table 2). After 16 h of topical application, the amount of percutaneously delivered B‐ACS was 12.7 ± 1.5 mg/kg. By contrast, when betulin was directly dissolved and applied in the O/W cream formulation, only 2.14 ± 0.21 mg/kg was delivered. B‐ACS significantly enhanced betulin delivery compared with simple application in the formulation, outperforming typical liposomal systems which often achieve ∼80% encapsulation and lower permeation for similar actives. [30]

**TABLE 1:** Characterization of arginine‐caprylate self‐assembly (ACS) and betulin‐loaded ACS.

Sample	Size (nm)	PDI	Zeta potential (mV)	Encapsulation efficacy (%)
ACS	4.7 ± 0.1	0.007	−9.98	—
B‐ACS	236.9 ± 10.3	0.257	−9.91	98.3 ± 1.3

**TABLE 2:** Cumulative amount of betulin permeated into the dermis through 1‐mm‐thick porcine skin over 16 h. Values are means ± standard deviation (*n* = 3).

Sample	Cumulative retention amount for 16 h [mg/kg]	Transdermal efficacy (%)
Cream with betulin	2.14 ± 0.21	3.57 ± 0.35
Cream with B‐ACS	12.7 ± 1.5	21.2 ± 2.5

**FIGURE 3:**
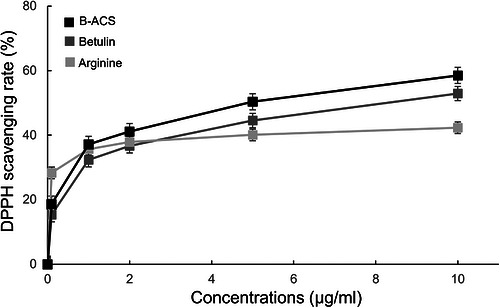
Activities of arginine, betulin, and B‐ACS via DPPH radical scavenging activity.

Arginine and betulin function as antioxidants with free radical‐scavenging activity. Confirming whether betulin‐loaded B‐ACS exhibits antioxidant efficacy is essential. The antioxidant activities of arginine, betulin, and B‐ACS were evaluated based on their ability to scavenge DPPH free radicals (Figure 3), with IC50 values of >10 ppm (no 50% reached) for arginine, 8.26 ppm for betulin, and 4.87 ppm for B‐ACS. Arginine showed 28.34% scavenging ability at the lowest tested dose (0.1 ppm), indicating high efficacy at low concentrations. Betulin and B‐ACS exhibited scavenging abilities of 15.36% and 18.66%, respectively. However, as the treatment concentration increased to 2, 5, and 10 ppm, arginine reached saturation. By contrast, the scavenging abilities of betulin and B‐ACS continued to increase up to the maximum treatment concentration (10 ppm). In particular, B‐ACS achieved 58.33% scavenging ability at 10 ppm. These findings suggest that arginine may serve as a carrier for betulin, and the combination of these two components could enhance antioxidant efficacy for skin applications. The in vitro results support the use of B‐ACS in vivo, not only for improving skin delivery efficiency but also for enhancing antioxidant effectiveness.

### 3.3 In Vitro Inhibitory Effect of UV‐Induced Inflammation and Skin Aging Related Factors

The inhibitory effects of B‐ACS were compared with those of arginine, caprylic acid, and betulin on the expression of seven inflammatory factors induced by UVB exposure. Since TNF‐*α* and IL‐6 mediate immune responses and MCP‐1 and IL‐8 activate inflammatory cells, their overexpression may lead to worsening of skin conditions. Excessive secretion of IL‐4 causes persistent inflammation and impairs skin barrier function. In addition, MMP‐1 and MMP‐9 degrade collagen and gelatin, respectively. These factors are expressed in keratinocytes upon UVB exposure, and UV‐induced inflammation promotes photoaging, leading to uneven skin tone, loss of elasticity, and increased wrinkles.

**FIGURE 4:**
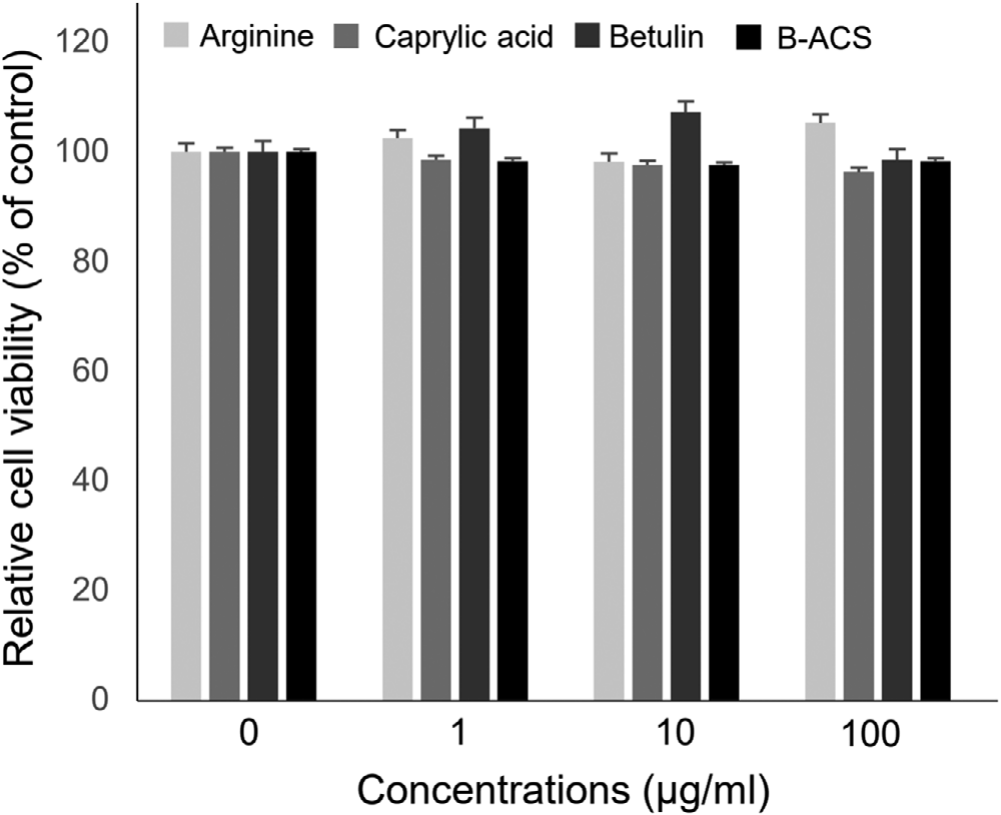
Evaluation of cellular cytotoxicity of arginine, caprylic acid, betulin, and B‐ACS at 0, 1, 10, and 100 ppm.

**FIGURE 5:**
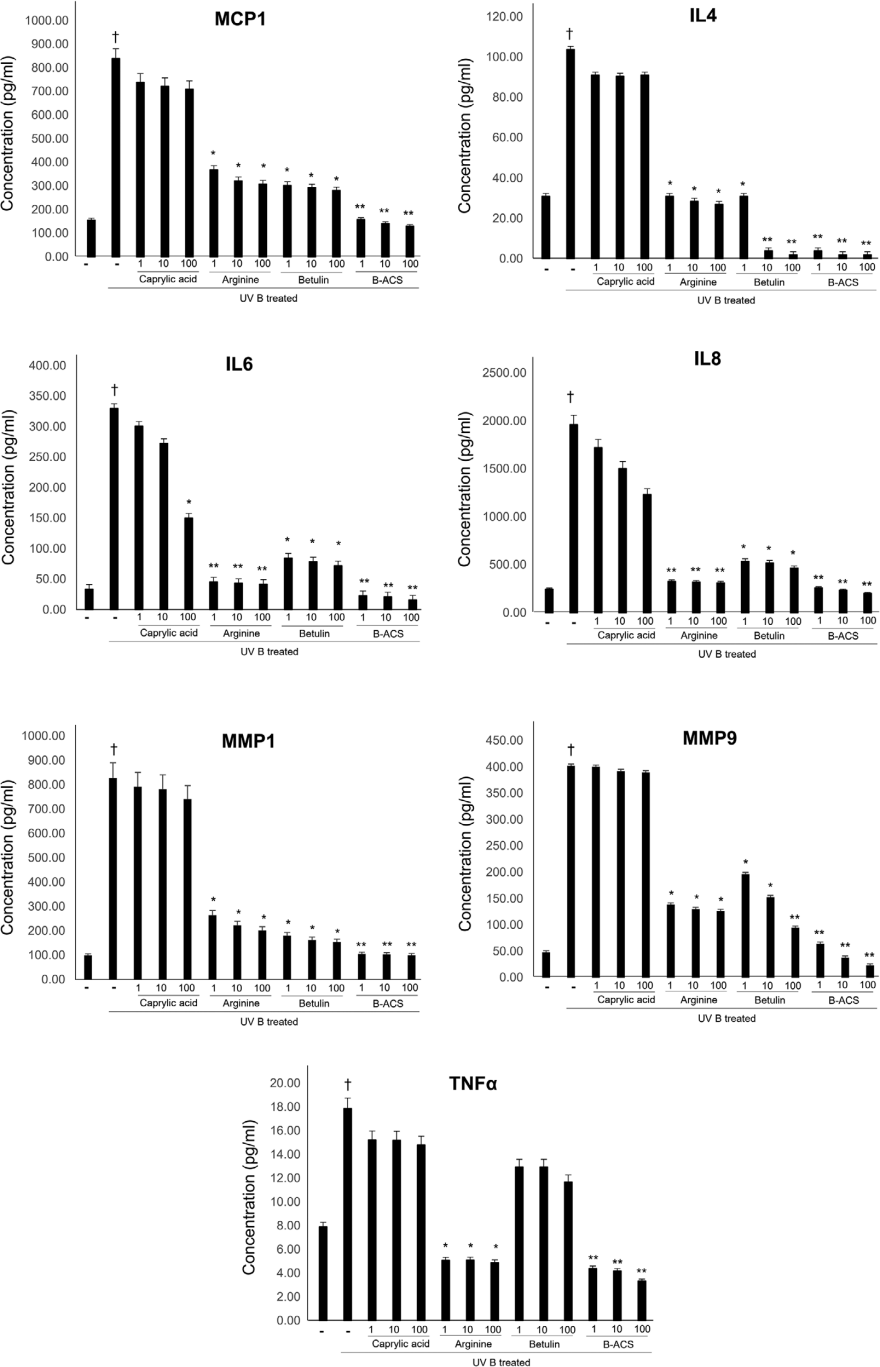
Results of the inhibitory effects of caprylic acid, arginine, betulin, and B‐ACS after UVB irradiation in HaCaT cells on the expression of MCP, IL4, IL6, IL8, MMP1, MMP9, TNF‐*α*. Each column presents the mean ± SD. † indicates a significant difference before and after UVB treatment at *p* < 0.05. * or ** indicates a significant difference between the groups depending on the presence or absence of agent treatment under UVB treatment conditions at *p* < 0.05 or *p* < 0.01, respectively.

Before cell treatment, the WST‐1 assay confirmed that arginine, caprylic acid, betulin, and B‐ACS did not cause cytotoxicity, even at the maximum treatment concentration (Figure 4; quantitative data in Table 3). The degree of inhibition of these factors after treatment with 1, 10, and 100 ppm of keratinocytes and subsequent UVB irradiation is shown in Figure 5 (quantitative percentages in Table 4). Although caprylic acid did not show a significant inhibitory effect at the treatment concentration, arginine, betulin, and B‐ACS, previously evaluated for their antioxidant activities, showed significant inhibition of most factors. Notably, inhibition of MMP, which is directly related to elasticity loss, showed the most pronounced improvement. B‐ACS (100 ppm) improved MMP‐1 inhibition by 8.17%/16.33% compared with arginine (100 ppm) and betulin (100 ppm), while MMP‐9 inhibition showed an even more remarkable improvement of 23.33%/37.46%. The inhibitory effects of arginine and betulin on inflammation‐related factors varied depending on the type. However, the combined application of arginine and betulin in B‐ACS showed a dramatic increase in efficacy in all evaluated factors (*p* < 0.01). All cytokine and MMP values were normalized to the UVB‐exposed no‐treatment control group (not baseline). B‐ACS (100 ppm) provided quantitatively superior inhibition compared with arginine or betulin alone (e.g., 8.17% and 23.33% greater reduction in MMP‐1 and MMP‐9 expression, respectively; see Table 4 for full quantitative data). While HaCaT cells provide a reproducible model for UV responses, they may not fully recapitulate primary keratinocyte behavior, such as differentiation and apoptosis regulation, potentially underestimating effects; this limitation is addressed by our complementary artificial skin and clinical validations.

**TABLE 3:** Quantitative data corresponding to Figure 4.

Concentrations (µg/ml)	Relative viability (% of control)			
	Arginine	Caprylic acid	Betulin	B‐ACS
0	100.0 ± 2.2	100.0 ± 1.3	100.0 ± 2.7	100.0 ± 1.1
0.1	102.5 ± 2.5	98.5 ± 1.4	104.3 ± 3.1	98.3 ± 1.2
1	98.2 ± 1.9	97.6 ± 1.6	107.2 ± 3.3	97.6 ± 1.2
10	105.3 ± 1.8	96.3 ± 1.6	98.5 ± 2.9	98.3 ± 1.3

**TABLE 4:** Quantitative data corresponding to Figure 5.

		Conc. (µg/ml)	Cytokines & MMPs (pg/ml)						
			MCP1	IL4	IL6	IL8	MMP1	MMP9	TNF*α*
UVB‐	No treatment	0	156.30 ± 7.8	30.4 ± 0.4	33.4 ± 2.1	246.6 ± 12.4	99.7 ± 7.6	47.8 ± 1.2	7.9 ± 0.4
UVB+	No treatment	0	839.8 ± 44.5	103.9 ± 1.3	330.9 ± 16.2	1963.8 ± 96.4	828.8 ± 64.5	402.7 ± 8.6	17.8 ± 0.9
	Caprylic acid	1	739.4 ± 37.6	91.1 ± 1.1	301.7 ± 15.7	1722.2 ± 86.2	792.2 ± 60.6	400.7 ± 8.4	15.5 ± 0.7
	Caprylic acid	10	722.1 ± 31.1	90.8 ± 0.9	272.8 ± 12.6	1053.8 ± 52.9	782.7 ± 58.4	392.6 ± 8.6	15.2 ± 0.7
	Caprylic acid	100	710.1 ± 31.6	91.3 ± 0.8	150.1 ± 7.5	1233.7 ± 65.4	741.1 ± 48.6	390.1 ± 7.6	14.8 ± 0.4
	Arginine	1	368.4 ± 28.6	30.3 ± 0.4	45.6 ± 3.7	326.3 ± 16.2	264.9 ± 17.8	138.7 ± 2.4	5.0 ± 0.1
	Arginine	10	322.1 ± 22.5	28.1 ± 0.3	43.8 ± 3.4	319.5 ± 14.5	223.3 ± 15.2	130.2 ± 2.2	5.1 ± 0.1
	Arginine	100	309.1 ± 20.6	26.6 ± 0.2	41.8 ± 3.1	306.2 ± 12.6	202.6 ± 15.1	126.6 ± 2.3	4.8 ± 0.1
	Betulin	1	303.2 ± 20.1	30.5 ± 0.4	84.4 ± 5.2	536.9 ± 22.6	180.1 ± 12.3	196.6 ± 1.9	12.9 ± 0.1
	Betulin	10	293.5 ± 17.8	4.1 ± 0.4	78.4 ± 4.6	520 ± 23.5	162.3 ± 11.6	152.4 ± 1.4	12.9 ± 0.1
	Betulin	100	281.8 ± 14.6	2.6 ± 0.2	72.6 ± 3.8	465.4 ± 20.1	155.5 ± 10.5	94.8 ± 1.8	11.6 ± 0.1
	B‐ACS	1	158.7 ± 8.9	4.0 ± 0.2	23.3 ± 1.6	258.3 ± 16.5	105.1 ± 7.8	64.2 ± 1.6	4.4 ± 0.1
	B‐ACS	10	141.7 ± 7.6	2.3 ± 0.1	21.7 ± 1.2	234.7 ± 12.2	103.8 ± 6.5	37.9 ± 1.4	4.2 ± 0.0
	B‐ACS	100	130.7 ± 6.9	2.1 ± 0.1	16.5 ± 1.1	202.1 ± 10.6	100.1 ± 6.2	22.7 ± 1.1	3.3 ± 0.0

**FIGURE 6:**
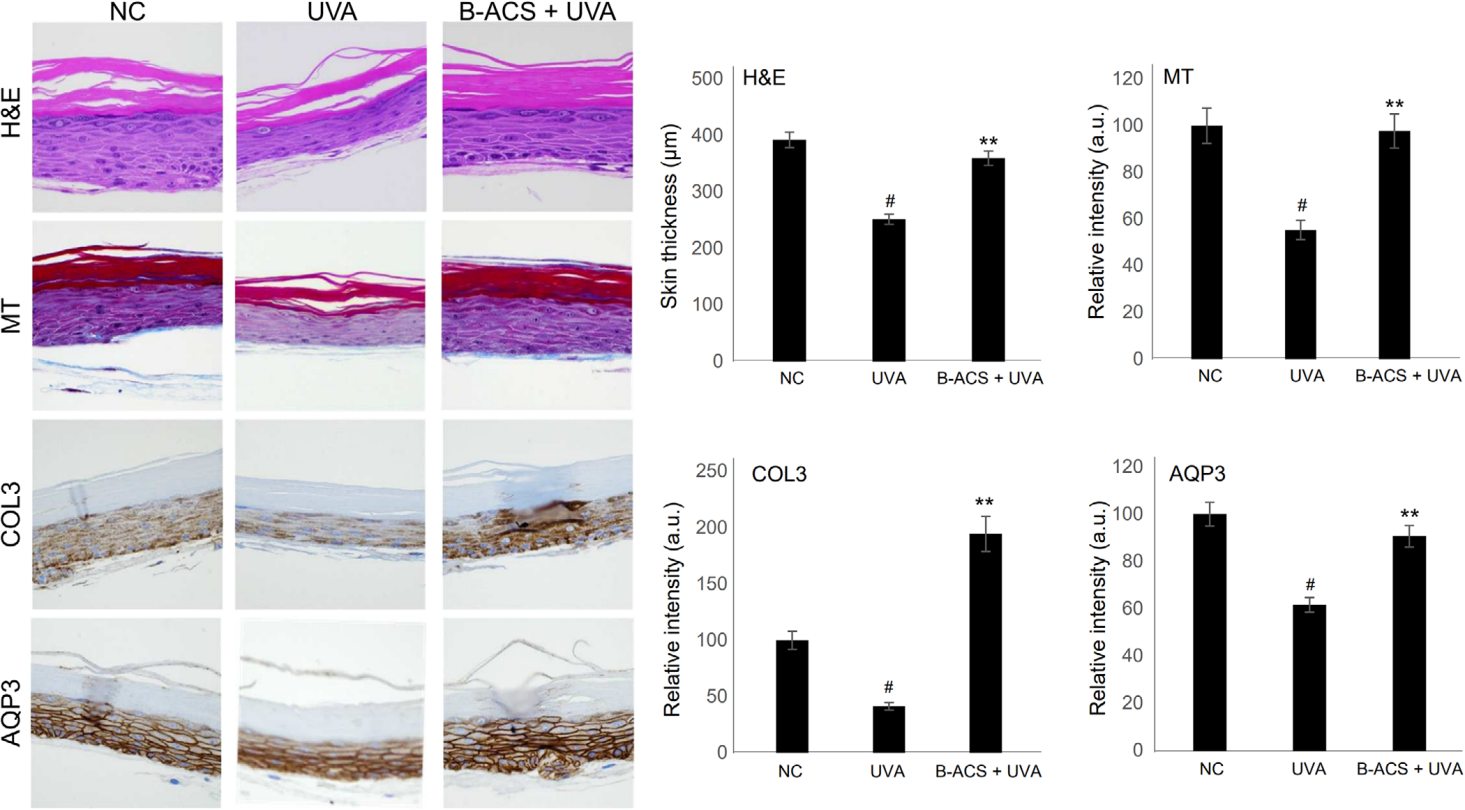
Effect of UVA on B‐ACS treatment on KeraSkin^TM^ artificial skin. Results of H&E staining, Masson's trichrome (MT) staining, and immunostaining using COL3 and AQP3 antibodies, comparison of skin thickness, comparison of collagen contents by MT staining, comparison of expression levels by COL3 immunostaining, comparison of expression levels by AQP3 immunostaining. Each column represents untreated, UVA‐treated, and B‐ACS + UVA‐treated samples, and represents the mean ± SD. # indicates a significant difference before and after UVA treatment at *p* < 0.05. ** indicates a significant difference between the groups depending on the presence or absence of drug treatment under UVA treatment conditions at *p* < 0.01.

Before clinically confirming the efficacy of UV irradiation in inhibiting photoaging, the antiaging effects of B‐ACS were evaluated by irradiating a reconstructed human skin model (Keraskin) to highly skin‐penetrating UVA. Although UVB has higher energy, 95% of the UV rays that reach the earth's surface are UVA, which penetrates the skin more deeply and contributes significantly to photoaging. Changes in Keraskin treated with B‐ACS and irradiated with 0.75 J/cm^2^ of UVA were compared with untreated skin (negative control) and UVA‐only (Figure 6; quantitative data in Table 5). Representative original, unprocessed images of H&E staining, Masson's trichrome staining, COL3 immunostaining, and AQP3 immunostaining—maintained without contrast or brightness adjustments for objective comparison—are provided in Figure S2. No positive control (e.g., retinoic acid) was included to focus on B‐ACS specificity, though retinoic acid is effective in similar models. The average skin thickness decreased by 35.8% after UV irradiation. However, with B‐ACS pretreatment, the decrease was limited to 8.3%, preventing 42.9% of the thickness reduction observed in untreated skin. Masson's trichrome‐stained skin analysis using Image J confirmed that collagen levels (stained blue) were maintained 76.5% higher in the B‐ACS‐treated group than in the untreated group. In particular, COL3 showed a 3.7‐fold higher expression in the B‐ACS‐treated group than in the untreated group and a 94.5% increase compared with the UV‐untreated group. These results suggest that B‐ACS inhibits skin aging not only by inhibiting collagen degradation through its antioxidant properties but also by inducing additional synthesis of type III collagen (COL3, key for elasticity and matrix repair), though markers like TGF‐*β*1 or procollagen were not assessed—a limitation for future studies. To assess skin hydration, AQP3 expression was evaluated, revealing that B‐ACS treatment prevented 47.0% of UV‐induced damage, which is clinically meaningful for maintaining barrier function and reducing dryness. These results suggest that B‐ACS effectively inhibits UV‐induced inflammation and mitigates the subsequent skin aging process.

**TABLE 5:** Quantitative data corresponding to Figure 6.

	NC	UVA	B‐ACS + UVA
Skin thickness (µm)	392.5 ± 13.7	252.4 ± 8.9	360.0 ± 12.8
MT (relative intensity)	100.0 ± 7.5	55.4 ± 4.2	97.8 ± 7.4
COL3 (relative intensity)	100.0 ± 8.2	41.3 ± 3.3	194.5 ± 15.6
AQP3 (relative intensity)	100.0 ± 5.1	61.7 ± 3.1	90.7 ± 4.6

### 3.4 Clinical Inhibitory Efficacy of B‐ACS on UV and Heat‐Induced Aging

**FIGURE 7:**
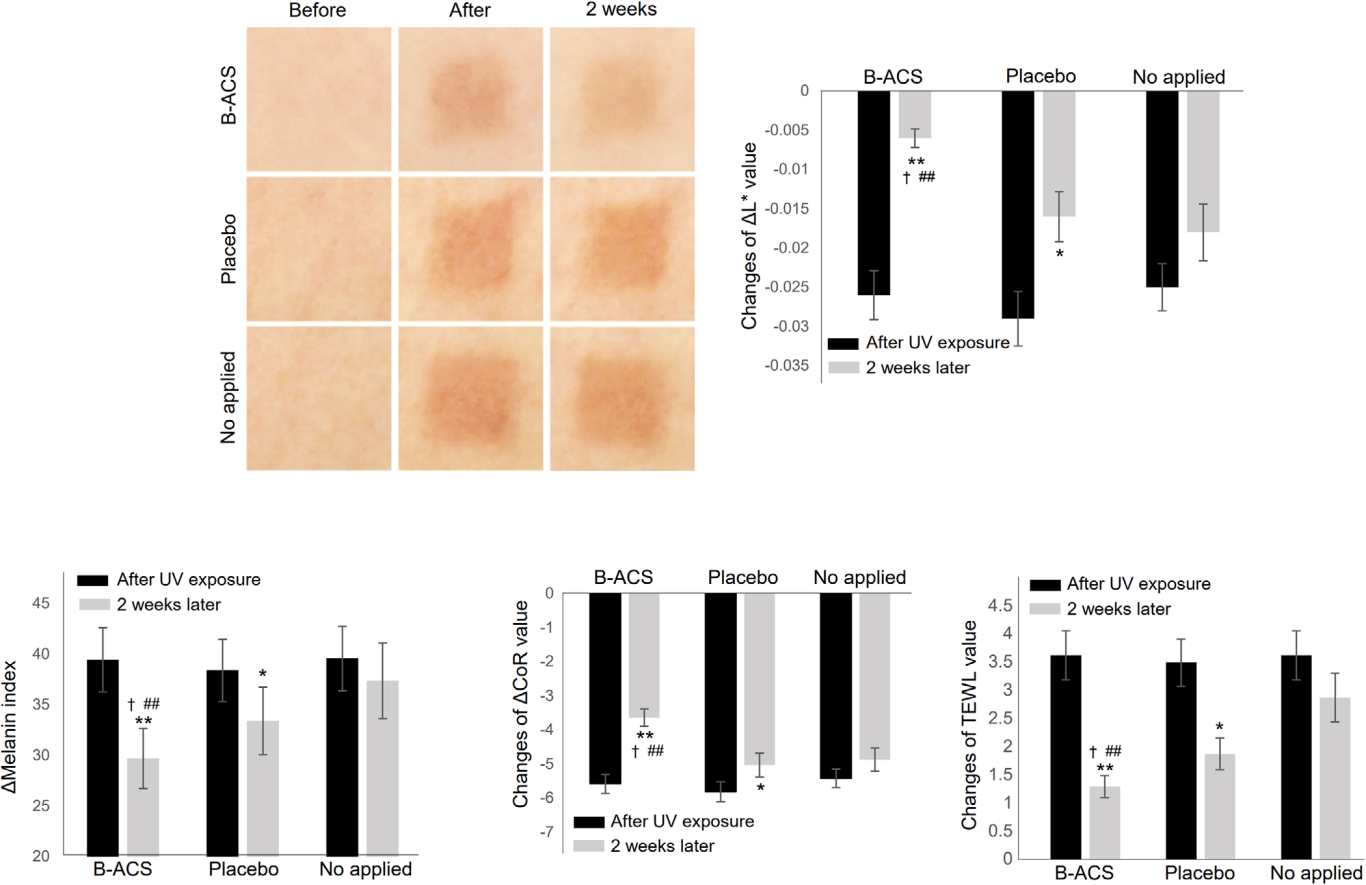
Images of changes in skin brightness, and changes of the skin brightness, pigmentation, skin elasticity, and TEWL evaluated in the application areas of cream containing 3% (w/w) B‐ACS, placebo cream, and no treatment for 2 weeks after UV ray irradiation. Each column represents the mean ± SD. * or ** indicates a significant difference between immediately after UV irradiation and 2 weeks later at *p* < 0.05 or *p* < 0.01, respectively, and †, ## indicate significant differences between the application areas of cream containing 3% (w/w) B‐ACS and the application areas of placebo cream or no treatment at *p* < 0.05 and *p* < 0.01, respectively.

A randomized, double‐blind, placebo‐controlled clinical evaluation was performed as specified in the Materials and Methods section. The changes in photoaged skin after irradiating with 76.75 mJ/cm^2^ of UVA+UVB are shown in Figure 7. Skin brightness (L value) increased significantly after 2 weeks of 3% (w/w) B‐ACS cream use compared with pre‐application levels (after UV irradiation). Brightness improvement at the B‐ACS site was 2.59‐fold higher than at the placebo site and 3.41‐fold higher than at the untreated site (*p* < 0.05). Pigmentation (melanin index) decreased significantly in both the B‐ACS and placebo groups, but the reduction was 1.91‐fold greater in the B‐ACS group than in the placebo group and 4.46‐fold greater than in the untreated group (*p* < 0.05). Skin elasticity (CoR value) increased significantly in both the B‐ACS and placebo groups, yet the improvement was 1.69‐fold greater in the B‐ACS group than in the placebo group and 2.90‐fold greater than in the untreated group (*p* < 0.05). Transepidermal water loss (TEWL) decreased 1.39‐fold more in the B‐ACS group than in the placebo group and 3.13‐fold more than in the untreated group (*p* < 0.05). All parameters were normalized to baseline (pre‐UV) values, with statistical significance determined by repeated‐measures ANOVA and post‐hoc Bonferroni correction.

Absolute baseline skin parameters were measured by ANTERA 3D (L value) and Mexameter (melanin index) prior to UV irradiation and were comparable across sites (full values in clinical report Appendix). Fold increases were calculated as (B‐ACS improvement % / placebo improvement %). The 2‐week observation period was chosen to capture initial antiaging effects, consistent with the epidermal turnover cycle and similar pilot cosmetic trials; longer‐term stability of improvements requires further confirmation.

Inter‐subject variability was low; all 12 participants demonstrated positive responses on B‐ACS sites (improvements >15% in at least three parameters). Demographic breakdown showed a mean age of 45.58 ± 12.57 years (range 25–66 years). Baseline skin parameters (L and melanin index measured by ANTERA 3D and Mexameter) were comparable across application sites. The study was conducted during winter (December 2024–February 2025) in Seoul to minimize natural UV exposure variability; participants were instructed to avoid sunscreen, functional cosmetics, and outdoor activities.

Possible placebo effects were observed for subjective parameters (e.g., brightness and elasticity), but objective instrumental measurements (ANTERA 3D, Ballistometer, Tewameter, Mexameter) and the heat‐aging index (carbonylated proteins) showed statistically superior improvements with B‐ACS versus placebo (*p* < 0.05), confirming the specific efficacy of the active ingredient. Confidence intervals were not calculated in the original statistical plan; results are presented as mean ± SD with *p*‐values from repeated‐measures ANOVA (Bonferroni‐corrected).

**FIGURE 8:**
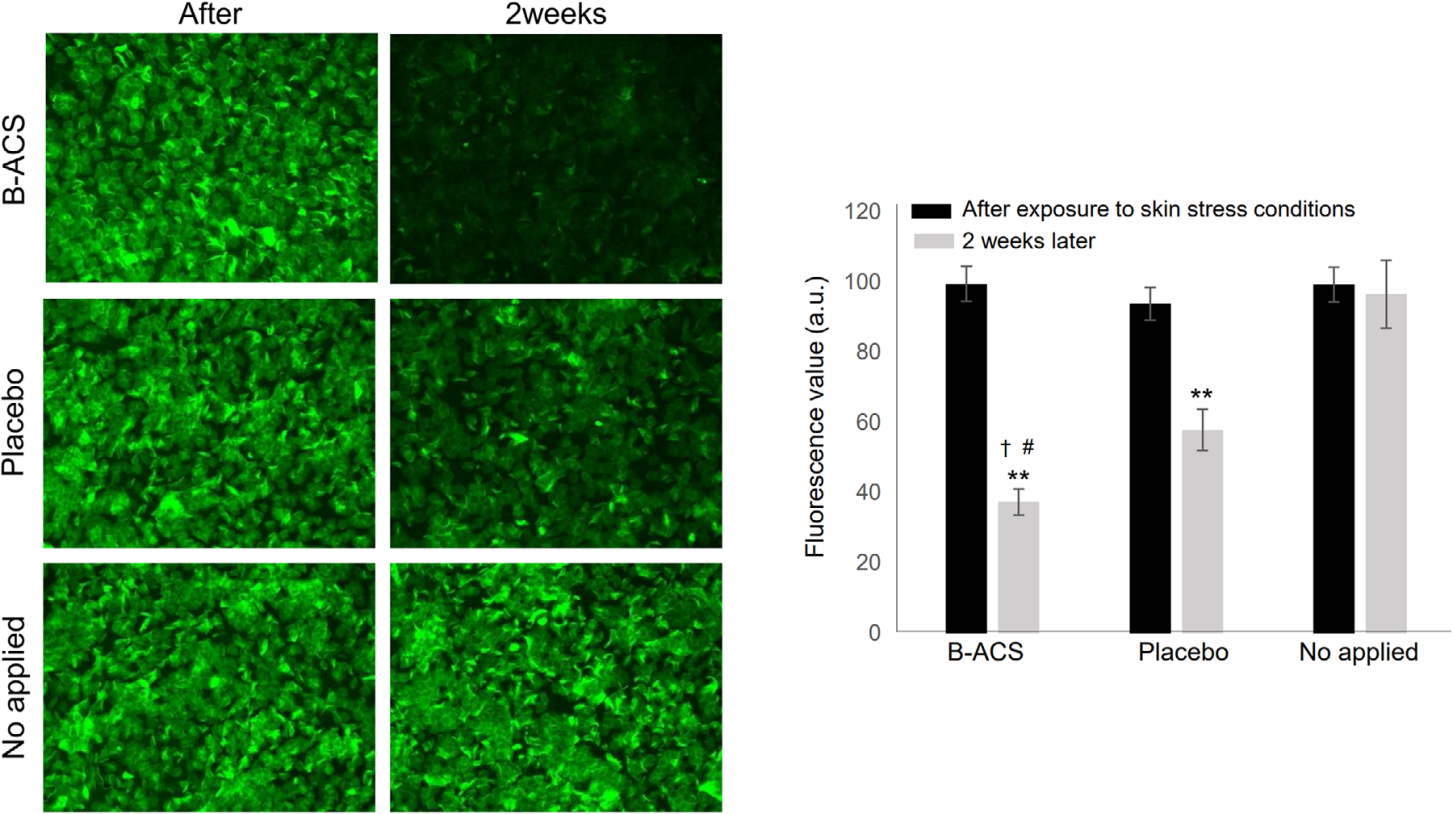
Results of changes in the ΔGreen value (carbonylated protein), an index of thermal aging in the keratinocytes, increased in a high‐temperature, dry environment due to infrared irradiation. Each column represents the mean ± SD. ** indicates a significant difference between immediately after UV irradiation and 2 weeks later at *p* < 0.01, and †, # indicate a significant difference between the areas where cream containing 3% (w/w) B‐ACS was applied and the areas where placebo cream was applied or no application at *p* < 0.05.

Recent studies have shown that overexposure to infrared rays generates ROS, triggers inflammation, and accelerates aging in a manner similar to UV radiation. Interest in and research on heat‐induced aging have been increasing. [31, 32] We evaluated the ability of B‐ACS to reduce thermal aging, specifically the increase in the thermal aging index (carbonylated protein) in the stratum corneum. A high‐temperature, dry environment (temperature: 40.00 ± 2°C, humidity: 22.00 ± 2%) was created using an infrared irradiator under controlled conditions (Figure 8). Results showed that after 20 min of exposure to a high‐temperature, dry environment, both the 3% (w/w) B‐ACS cream and placebo cream led to a statistically significant decrease in the thermal aging index after 2 weeks of use. However, the reduction was more pronounced in the area treated with B‐ACS cream, showing a significant improvement of 1.63 times compared with the placebo‐treated area and an 18.57 times compared with the untreated area.

## 4 Discussion

As average life expectancy continues to increase, maintaining skin health in old age has become increasingly important. However, increased average temperatures and prolonged UV exposure present significant challenges to preserving skin health and slowing the aging process. Chronic low‐level inflammation is a key contributor to skin aging, as persistent inflammation increases inflammatory cytokine levels, leading to cellular damage. In particular, UV rays that can induce inflammation through ROS generation have long been investigated as a major factor in extrinsic aging. However, recent studies have shown that infrared radiation and various heat sources also induce inflammation and promote aging, highlighting the need for skincare solutions that can mitigate heat‐induced aging. [33, 34]

Betulin has demonstrated remarkable antibacterial, anti‐inflammatory, and antioxidant properties. However, its industrial applications remain limited due to poor solubility in water and most solvents used in skincare products. To address this limitation, B‐ACS, a self‐assembled complex composed of arginine–caprylate ion pairs, was developed and loaded with betulin. This formulation was designed to suppress UV‐ or heat‐induced inflammation while preventing the deterioration of skin elasticity, barrier function, and pigmentation. These benefits are attributed to the antioxidant activity of B‐ACS, which exhibited better ROS scavenging activity compared with its individual antioxidant components, arginine and betulin, at the maximum treatment concentration (10 ppm) in this study. Treatment with B‐ACS effectively suppressed the expression of UVB‐activated inflammatory and aging‐related factors (MCP‐1, TNF‐*α*, IL‐4, IL‐6, IL‐8, MMP‐1, MMP‐9) in HaCaT cells. In particular, MCP‐1 and IL‐8, cytokines that mediate skin inflammation, were significantly reduced. In addition, MMP‐1 and MMP‐9, proteases that can directly induce skin barrier abnormalities, were inhibited. The suppression of these factors is directly related to alleviating UV‐ and heat‐induced aging, likely through B‐ACS's inhibition of NF‐κB signaling and apoptotic pathways (e.g., Fas/caspase‐8), reducing cytokine release and MMP activation. These effects were also validated in an artificial skin model irradiated with UVA, the predominant component of UV radiation reaching the surface and a major contributor to skin aging. UVA exposure caused a decrease in collagen content, including type III collagen, which is directly involved in skin elasticity, along with a reduction in aquaporin 3, which is involved in skin hydration. Skin thickness also decreased as a result of UVA exposure. However, B‐ACS prevented the loss of aquaporin 3 by 47.0% and notably induced type III collagen expression by 94.5% compared with pre‐irradiation levels. Consequently, B‐ACS prevented the decrease in skin thickness by 42.9%, maintaining it at a level similar to pre‐UVA exposure. These results were also observed in clinical evaluations involving combined UVA and UVB irradiation. A cream containing 3% (w/w) B‐ACS improved skin elasticity by 1.69‐fold and skin brightness and pigmentation by 2.59‐ and 1.91‐fold, respectively, compared with the placebo control group after 2 weeks of UV irradiation. Improvement in skin barrier was evaluated using TEWL measurements, and the results showed a 1.39‐fold improvement compared with the placebo control group. We created a heat‐aging environment using infrared rays in a controlled space. Heat‐aging inhibition was evaluated by analyzing changes in fluorescence intensity using carbonylated proteins in the stratum corneum as a heat‐aging index. A cream containing 3% (w/w) B‐ACS showed a 1.63‐fold improvement in the heat‐aging index compared with the placebo control group. B‐ACS reduces protein carbonylation likely by scavenging ROS to inhibit lipid peroxidation cascades and downregulating HSP70 induction, preventing oxidative protein modifications.

This mechanism is particularly relevant for heat‐aging protection, as carbonylated proteins serve as a direct marker of thermal protein damage; betulin's ROS‐scavenging activity, amplified by the B‐ACS carrier, likely attenuates HSP70‐mediated stress responses and subsequent protein carbonylation cascades.

B‐ACS exhibited notable ROS‐scavenging activity (IC50 = 4.87 ppm), which is comparable to values reported in literature for established topical antioxidants like vitamin C or resveratrol in similar settings. This high efficacy may be attributed to arginine's role in enhancing betulin delivery through cationic ion‐pairing for micelle stability, polyarginine motifs for stratum corneum disruption, and biological NO‐mediated absorption. While the 1.91‐fold reduction in pigmentation and 1.69‐fold improvement in elasticity observed in this study compare favorably to reported outcomes for retinol‐based creams or liposomal triterpenoids (∼1.2 to 1.5‐fold), and align with previous betulin studies in oleogels or nano‐sprays, it is essential to emphasize that no direct head‐to‐head comparative clinical trials were conducted under an identical protocol. [11, 35] Therefore, these external comparisons should be interpreted as supportive context rather than definitive evidence of clinical superiority.

This study was designed as a pilot trial with *n* = 12 participants. Although the sample size is small and limits statistical power and generalizability, it was sufficient to detect statistically significant differences (*p* < 0.05) versus placebo on multiple objective instrumental parameters. The observation period was limited to 14 days (consistent with the average epidermal turnover cycle of ∼28 days) to evaluate initial efficacy; longer‐term studies (≥8–12 weeks) are required to assess the durability of improvements. The study enrolled only female participants of Fitzpatrick skin types II–IV (Asian/Korean population), so results may vary across male populations, other ethnic groups, and different phototypes; future trials should include broader demographics.

The proposed mechanism integrates: Arginine‐caprylate self‐assembly solubilizes betulin for enhanced delivery; synergistic ROS scavenging reduces lipid peroxidation/carbonylation; this inhibits NF‐κB‐driven inflammation (cytokines/MMPs), preserving AQP3 hydration and upregulating COL3 via NO/TGF‐*β* for elasticity—interconnecting antioxidant, anti‐inflammatory, and reparative effects.

The HaCaT keratinocyte model and KeraSkinTM reconstructed human skin have inherent limitations: HaCaT cells are immortalized and lack full stratification and differentiation seen in primary keratinocytes, while the artificial skin model lacks vascular, immune, and appendage components. These constraints may underestimate or overestimate certain biological responses; however, the findings were corroborated by the randomized double‐blind human clinical trial, increasing translational relevance. Although the 2‐week duration demonstrated clear initial antiaging benefits (elasticity 1.69‐fold, brightness 2.59‐fold, pigmentation 1.91‐fold, TEWL reduction 1.39‐fold, heat‐aging inhibition 1.63‐fold versus placebo), longer‐term studies (≥8–12 weeks) are required before generalizing sustained cosmetic implications or recommending routine daily use. No skin irritation or adverse reactions were observed during the 2‐week clinical study. Although the surfactant‐free nature of the B‐ACS formulation is expected to provide a favorable long‐term safety profile, this pilot trial's short duration and small sample size limit definitive conclusions regarding chronic use. Therefore, cumulative irritation potential and long‐term tolerability must be evaluated in larger, extended‐duration studies (≥8–12 weeks). Such future research should specifically include sensitive‐skin cohorts and repeated daily application under real‐world conditions to confirm that B‐ACS remains a suitable and non‐irritating intervention for prolonged dermatological use.

Despite its findings, this study has several limitations. The 2‐week clinical duration was chosen for initial efficacy assessment, and the small sample size (*n* = 12) may constrain statistical power, though this was addressed by framing the work as a pilot study. Notably, the cohort consisted exclusively of female participants (aged 25–66 years) with Fitzpatrick skin types II–IV. This selection was a deliberate design choice to align with the primary target consumer group for the tested cosmetic product and to enhance data homogeneity by reducing inter‐gender variability in skin physiology and responses to UV and heat stress. However, this restricts the generalizability of the results to male populations and other ethnic groups. Additionally, while seasonal and dietary confounders were minimized by conducting the trial in winter with strict participant instructions, they were not entirely eliminated. Future research should involve longer observation periods and broader demographics to confirm sustained clinical efficacy.

## 5 Conclusion

B‐ACS enhances betulin's solubility/delivery, demonstrating superior ROS scavenging, inflammation suppression, and improvements in elasticity (1.69‐fold), brightness (2.59‐fold), pigmentation (1.91‐fold), TEWL (1.39‐fold), and heat‐aging (1.63‐fold) vs. placebo; limitations include short duration, female/Korean focus, small sample; future research should involve longer trials, diverse populations, and comparisons with standards like retinol. Despite these limitations, the efficacy of B‐ACS makes it an effective cosmetic ingredient in sunscreens or various skin care products.

## Conflicts of Interest

The authors declare no conflicts of interest.

## Ethics Statement

The study was approved by the Institutional Review Board of the Korea Institute of Dermatological Sciences (approval number: KIDS‐BDK097‐SNC). Written informed consent was obtained from all participants, and the study was conducted in accordance with the ethical principles of the Declaration of Helsinki and the Bioethics and Safety Act of the Republic of Korea.

## Funding Information

The authors have nothing to report.

## Supporting information



Supporting Information: srt70361‐sup‐0001‐figuresS1‐S2.pdf

Supporting Information: srt70361‐sup‐0002‐SuppMat.docx

## Data Availability

The data that support the findings of this study are available from the corresponding author upon reasonable request.

## References

[1] K. Nakai and D. Tsuruta , “What are Reactive Oxygen Species, Free Radicals, and Oxidative Stress in Skin Diseases?,” International Journal of Molecular Sciences 22, no. 19 (2021): 10799. 34639139 10.3390/ijms221910799 PMC8509443

[2] F. Flament , R. Bazin , S. Laquieze , V. Rubert , E. Simonpietri , and B. Piot , “Effect of the sun on Visible Clinical Signs of Aging in Caucasian Skin,” Clinical, Cosmetic and Investigational Dermatology 6 (2013): 221–232. 24101874 10.2147/CCID.S44686 PMC3790843

[3] O. Langselius , H. Rumgay , E. Vries , et al., “Global Burden of Cutaneous Melanoma Incidence Attributable to Ultraviolet Radiation in 2022,” International Journal of Cancer 157, no. 6 (2025): 1110–1119. 40421619 10.1002/ijc.35463

[4] D. M. Parkin , D. Mesher , and P. Sasieni , “13. Cancers Attributable to Solar (ultraviolet) Radiation Exposure in the UK in 2010,” British Journal of Cancer 105, no. 2 (2011): S66–S69. 22158324 10.1038/bjc.2011.486 PMC3252056

[5] M. Wei , X. He , N. Liu , and H. Deng , “Role of Reactive Oxygen Species in Ultraviolet‐induced Photodamage of the Skin,” Cell Division 19, no. 1 (2024): 1. 38217019 10.1186/s13008-024-00107-z PMC10787507

[6] P. Montero , I. Roger , J. Milara , and J. Cortijo , “Damaging Effects of UVA, Blue Light, and Infrared Radiation: *In Vitro* Assessment on a Reconstructed Full‐Thickness Human Skin,” Frontiers in Medicine 10 (2023): 1267409. 38105899 10.3389/fmed.2023.1267409 PMC10722227

[7] X. Hu , M. Chen , J. Nawaz , and X. Duan , “Regulatory Mechanisms of Natural Active Ingredients and Compounds on Keratinocytes and Fibroblasts in Mitigating Skin Photoaging,” Clinical, Cosmetic and Investigational Dermatology 17 (2024): 1943–1962. 39224224 10.2147/CCID.S478666 PMC11368101

[8] G. A. Tolstikov , O. B. Flekhter , E. E. Shultz , L. A. Baltina , and A. G. Tolstikov , “Betulin and Its Derivatives. Chemistry and Biological Activity,” Chemistry for Sustainable Development 13 (2005): 1–29.

[9] C. A. Dehelean , C. Soica , I. Ledeţi , et al., “Study of the Betulin Enriched Birch Bark Extracts Effects on Human Carcinoma Cells and Ear Inflammation,” Chemistry Central Journal 6 (2012): 137. 23158079 10.1186/1752-153X-6-137 PMC3527166

[10] W. Zhanga , H. Jianga , J. Yanga , et al., “Safety Assessment and Antioxidant Evaluation of Betulin by LC‐MS Combined With Free Radical Assays,” Analytical Biochemistry 587 (2019): 113460. 31563442 10.1016/j.ab.2019.113460

[11] X. Pan , Z. Zhong , X. Hu , et al., “Application of Nanotechnology in Anti‐Aging Cosmetics: Advantages, Challenges, and Prospects,” Polymer Bulletin 82 (2025): 8635–8725.

[12] A. Akbarzadeh , R. Rezaei‐Sadabady , S. Davaran , et al., “Liposome: Classification, Preparation, and Applications,” Nanoscale Research Letters 8 (2013): 102. 23432972 10.1186/1556-276X-8-102 PMC3599573

[13] A. G. Uriostegui‐Pena , A. Torres‐Copado , A. Ochoa‐Sanchez , G. Luna‐Bárcenas , P. Sahare , and S. Paul , “Nanoformulated Phytochemicals in Skin Anti‐Aging Research: An Updated Mini Review,” 3 BioTechniques 15 (2025): 31. 10.1007/s13205-024-04197-y PMC11699038 39760004

[14] A. L. Fameau and T. Zemb , “Self‐Assembly of Fatty Acids in the Presence of Amines and Cationic Components,” Advances in Colloid and Interface Science 207 (2013): 43–64. 24345730 10.1016/j.cis.2013.11.017

[15] Y. Altay , S. Cao , H. Che , L. K. E. A. Abdelmohsen , and J. C. M. van Hest , “Adaptive Polymeric Assemblies for Applications in Biomimicry and Nanomedicine,” Biomacromolecules 20, no. 11 (2019): 4053–4064. 31642319 10.1021/acs.biomac.9b01341 PMC6852094

[16] Q. Pan , S. Zhang , X. Yan , J. Guo , B. Li , and Y. Ping , “A Self‐Assembled Nano‐Spray Formulation for Synergistic Therapy of Anti‐Inflammation and Skin Repair,” Asian Journal of Pharmaceutical Sciences 20, no. 5 (2025): 101067. 41809259 10.1016/j.ajps.2025.101067 PMC12538012

[17] L. Nie , Y. Li , J. Xie , and S. Lu , “Arginine as a Promising Amino Acid for Functionalized Nanosystems: Innovations, Challenges, and Future Directions,” Nanotechnology Reviews 14, no. 1 (2025): 20250162.

[18] G. H. Li , Y. L. Liu , W. L. Xu , A. X. Song , and J. C. Hao , “Transition of Phase Structures in Mixtures of Lysine and Fatty Acids,” Journal of Physical Chemistry B 118 (2014): 14843–14851. 25469553 10.1021/jp510747y

[19] A. L. Fameau , B. Houinsou‐Houssou , J. L. Ventureira , et al., “Self‐Assembly, Foaming, and Emulsifying Properties of Sodium Alkyl Carboxylate/Guanidine Hydrochloride Aqueous Mixtures,” Langmuir 27 (2011): 4505–4513. 21405069 10.1021/la2002404

[20] K. C. Kwon , M. J. Kim , and S. A. Yoon , “Nanovesicles for Sensitive Skin Care Developed via Self‐Assembly of Glutamine Linoleate,” Journal of Cosmetic Dermatology 24, no. 4 (2025): e70195. 40259621 10.1111/jocd.70195 PMC12012325

[21] Y. Kang , X. Y. Tang , Z. G. Cai , and X. Zhang , “Supra‐Amphiphiles for Functional Assemblies,” Advanced Functional Materials 26 (2016): 8920–8931.

[22] P. P. Shah , P. R. Desai , D. Channer , and M. Singh , “Enhanced Skin Permeation Using Polyarginine Modified Nanostructured Lipid Carriers,” Journal of Controlled Release 161, no. 3 (2012): 735–745. 22617521 10.1016/j.jconrel.2012.05.011 PMC3412947

[23] R. Neupane , S. H. S. Boddu , J. Renukuntla , R. J. Babu , and A. K. Tiwari , “Alternatives to Biological Skin in Permeation Studies: Current Trends and Possibilities,” Pharmaceutics 12, no. 2 (2020): 152. 32070011 10.3390/pharmaceutics12020152 PMC7076422

[24] İ. Gulcin and S. H. Alwasel , “DPPH Radical Scavenging Assay,” Processes 11 (2023): 2248.

[25] J. C. Stockert , R. W. Horobin , and L. L. Colombo , “Blázquez‐Castro A, Tetrazolium Salts and Formazan Products in Cell Biology: Viability Assessment, Fluorescence Imaging, and Labeling Perspectives,” Acta Histochemica 120, no. 3 (2018): 159–167. 29496266 10.1016/j.acthis.2018.02.005

[26] J. Wang , H. Qiu , Y. Xu , et al., “The Biological Effect of Recombinant Humanized Collagen on Damaged Skin Induced by UV‐Photoaging: An *In Vivo* Study,” Bioact Mater 11 (2022): 154–165. 34938920 10.1016/j.bioactmat.2021.10.004 PMC8665261

[27] J. H. Oh , F. Karadeniz , J. I. Lee , Y. Seo , and C. S. Kong , “Protective Effect of 3,5‑Dicaffeoyl‑Epi‑Quinic Acid Against UVB‑Induced Photoaging in Human HaCaT Keratinocytes,” Molecular Medicine Reports 20 (2019): 763–770. 31115540 10.3892/mmr.2019.10258

[28] M. F. Cervantes Recalde , J. Schmidt , C. Girardi , et al., “Capsaicin Attenuates the Effect of Inflammatory Cytokines in a HaCaT Cell Model for Basal Keratinocytes,” Frontiers in pharmacology 15 (2024): 1474898. 39469627 10.3389/fphar.2024.1474898 PMC11513304

[29] A. Date , T. Shimakura , M. Sasaki , and M. Yamaguchi , “An Analytical Technique for Measuring Protein Carbonyl in the Stratum Corneum Using Surface Plasmon Resonance,” International Journal of Cosmetic Science 34, no. 1 (2011): 81–85. 21923732 10.1111/j.1468-2494.2011.00684.x

[30] Y. Liu , D. Gao , X. Zhang , et al., “Antitumor Drug Effect of Betulinic Acid Mediated by Polyethylene Glycol Modified Liposomes,” Materials Science and Engineering: C 64 (2016): 124–132. 27127036 10.1016/j.msec.2016.03.080

[31] M. Y. Akhalaya , G. V. Maksimov , A. B. Rubin , J. Lademann , and M. E. Darvin , “Molecular Action Mechanisms of Solar Infrared Radiation and Heat on human Skin,” Ageing Research Reviews 16 (2014): 1–11. 24742502 10.1016/j.arr.2014.03.006

[32] S. Cho , M. H. Shin , Y. K. Kim , et al., “Effects of Infrared Radiation and Heat on Human Skin Aging *In Vivo* ,” Journal of Investigative Dermatology Symposium Proceedings 14 (2009): 15–19. 19675547 10.1038/jidsymp.2009.7

[33] S. M. Pilkington , S. Bulfone‐Paus , C. E. M. Griffiths , and R. E. B. Watson , “Inflammaging and the Skin,” Journal of Investigative Dermatology 141 (2021): 1087–1095. 33358020 10.1016/j.jid.2020.11.006

[34] Y. Wang , L. Jiang , C. Weic , and H. Zhang , “Phase Behaviors and Self‐Assembled Properties of Ion‐Pairing Amphiphile Molecules Formed by Medium‐Chain Fatty Acids and L‐Arginine Triggered by External Conditions,” New Journal of Chemistry 41 (2017): 14486–14497.

[35] L. Lin , X. Chen , C. Liu , et al., “Comparative Efficacy of Topical Interventions for Facial Photoaging: A Network Meta‐Analysis,” Scientific Reports 15 (2025): 26889. 40707570 10.1038/s41598-025-12597-0 PMC12289910

