# Supplementary material for: Reduction of Ultraviolet‐ and Heat‐Induced Aging Using Betulin‐Loaded Arginine–Caprylate Self‐Assembly: Randomized Double‐Blind Clinical Trials
Source: Skin Res Technol. 2026 May 23;32(5):e70361. doi: 10.1111/srt.70361 (PMC13240441; doi:10.1111/srt.70361)
Supplement: Supplementary file 1 — Supporting Information: srt70361‐sup‐0001‐figuresS1‐S2.pdf [file SRT-32-e70361-s002.pdf]

Supplementary Figure

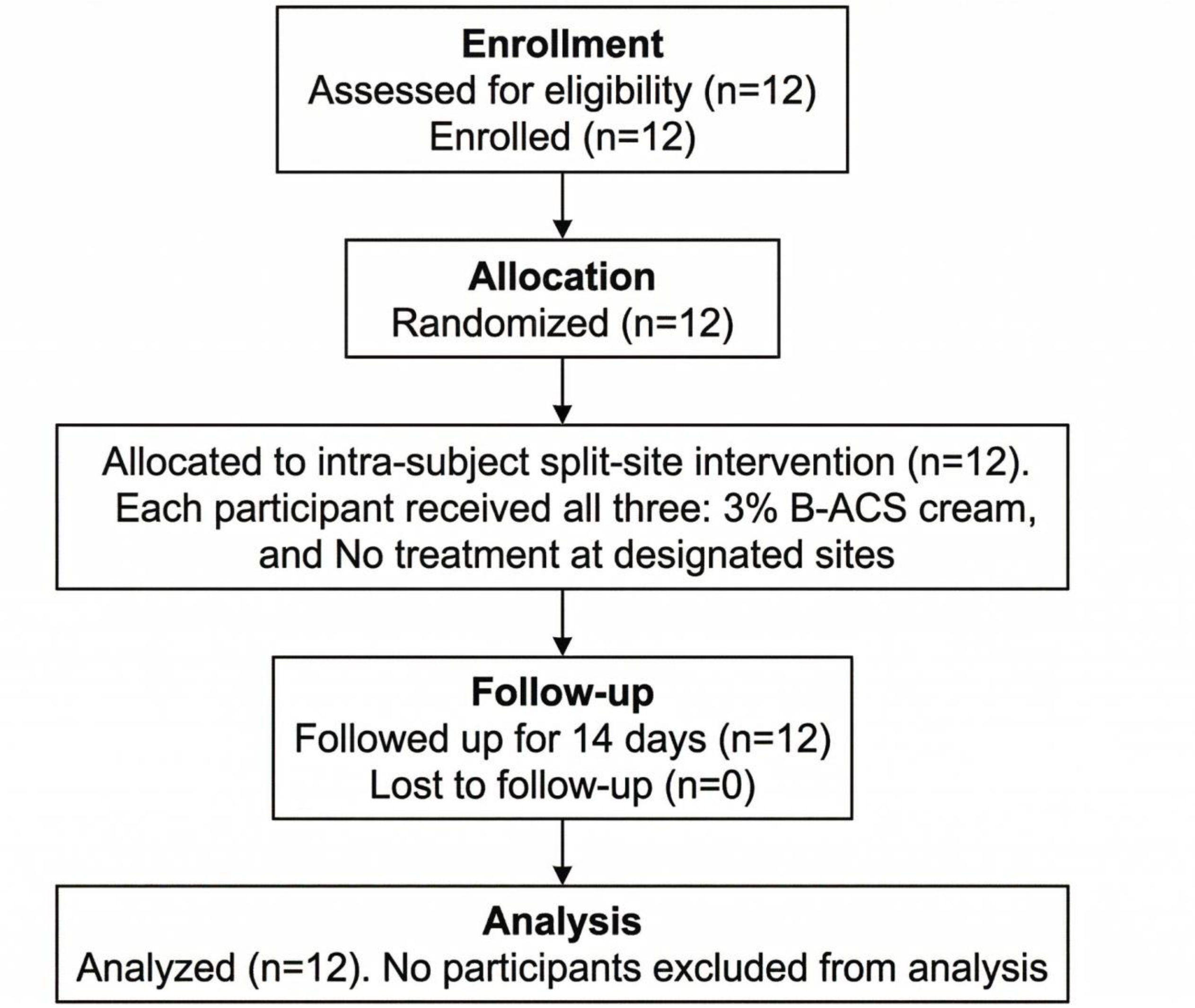

Supplementary Figure S1. CONSORT flow diagram of participant recruitment, randomization, and analysis. The study followed a randomized, double-blind, placebo-controlled, intra-subject split-site design. A total of 12 female participants (aged 25–66 years; mean 45.58 ± 12.57) who met the inclusion criteria (Fitzpatrick skin types II–IV) were enrolled and randomized. Randomization was performed using computer-generated block randomization (block size 3) to assign the three treatment sites (3% B-ACS cream, placebo cream, and untreated) on each participant's arm. All 12 participants completed the 2-week study period with no drop-outs or protocol deviations. All enrolled individuals were included in the final objective instrumental analysis (n = 12).

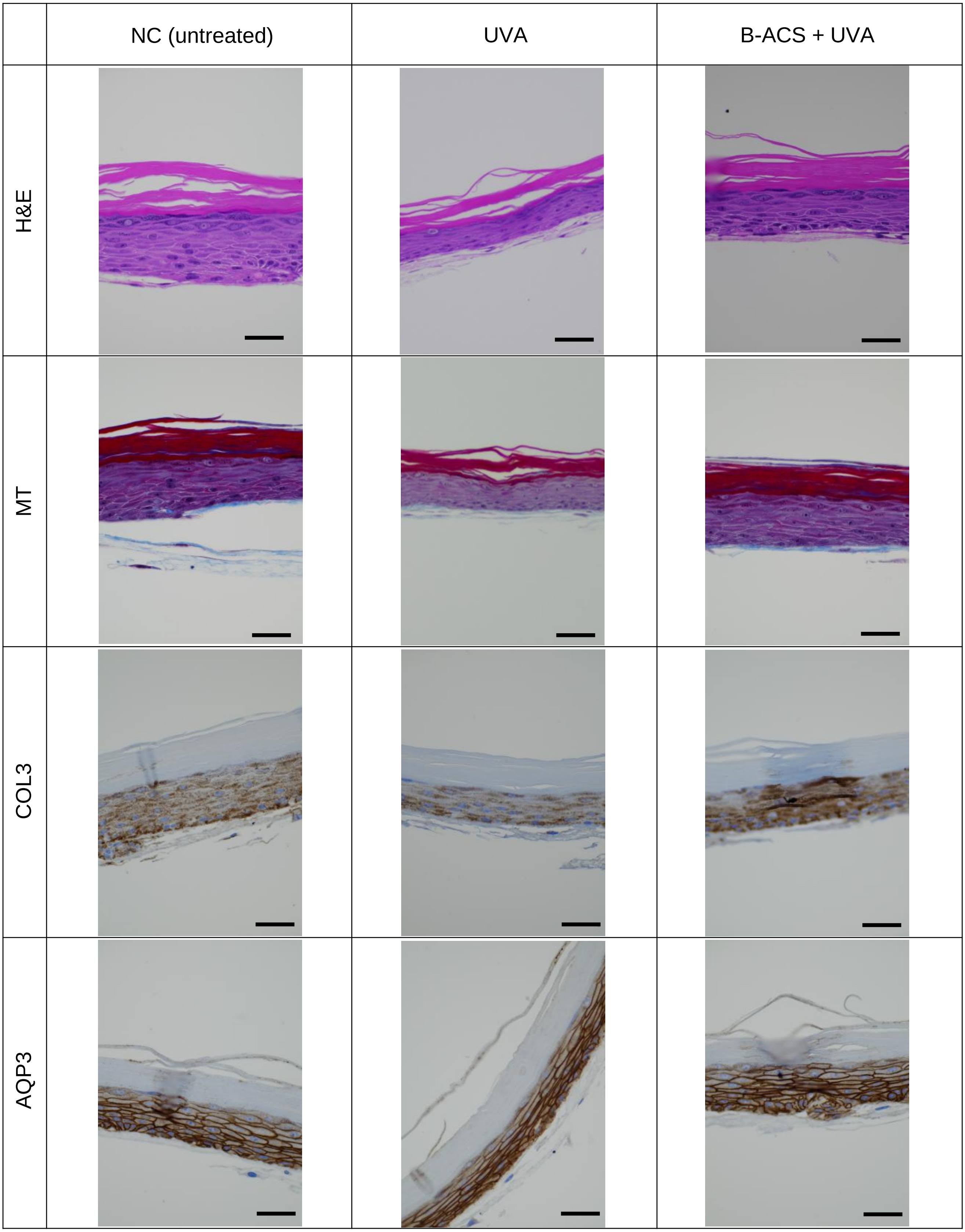

Supplementary Figure S2. Original raw images of Figure 6 (representative histological images of KeraSkin™ reconstructed human skin models). Whole-tissue sections stained with H&E and Masson’s trichrome, and immunostained for COL3 and AQP3 are shown to provide an expanded view of the morphological and biomolecular changes induced by UVA and the protective effects of B-ACS. Scale bars = 100 μm.
