# Supplementary material for: Reduction of Ultraviolet‐ and Heat‐Induced Aging Using Betulin‐Loaded Arginine–Caprylate Self‐Assembly: Randomized Double‐Blind Clinical Trials
Source: Skin Res Technol. 2026 May 23;32(5):e70361. doi: 10.1111/srt.70361 (PMC13240441; doi:10.1111/srt.70361)

**Supplementary Informations**

Informations about the institution for FCM production

- Information about the ethics committee for the production of FCMs for research based on micropigs: https://www.apures.com/company/

- Information about ethics: https://www.apures.com/rndsolution-ethics/

- Animal Experiment Plan Approval Form for FCM Production


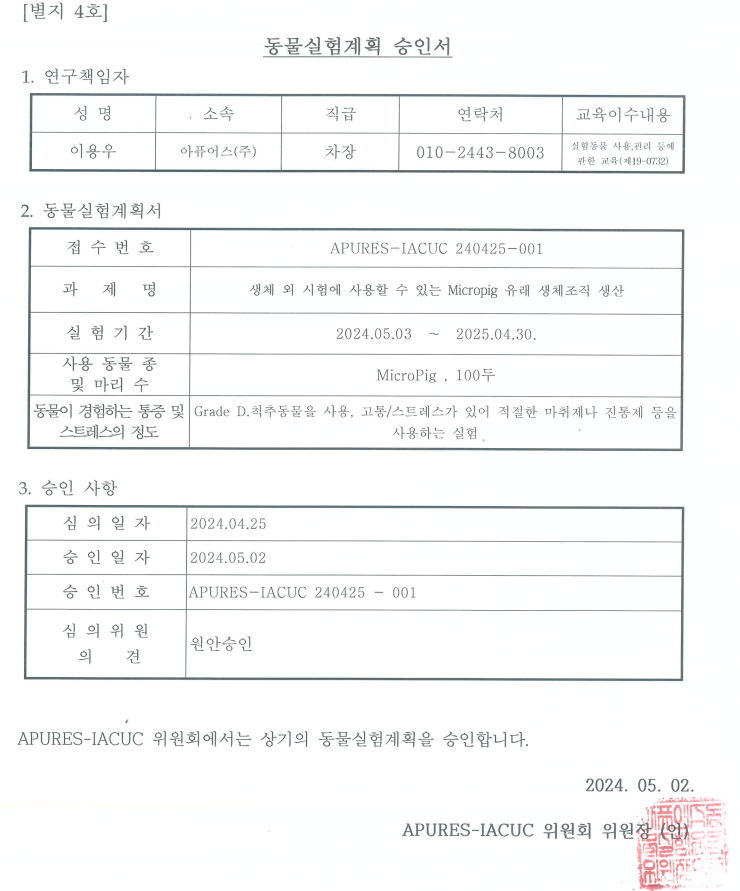


- Certificate for animal testing facility registration
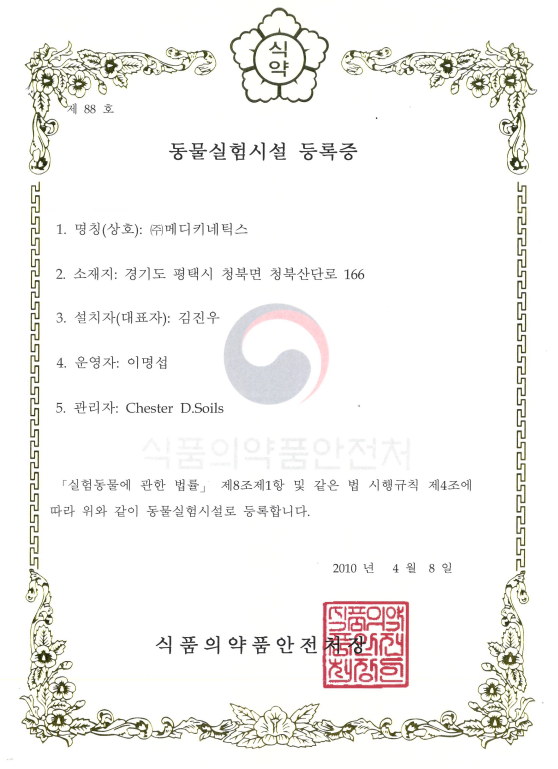


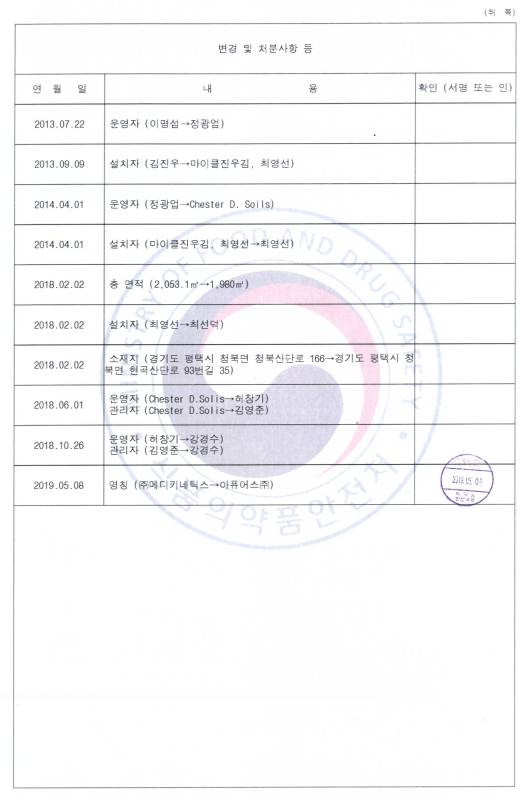

Supplement: Supplementary file 2 — Supporting Information: srt70361‐sup‐0002‐SuppMat.docx [file SRT-32-e70361-s001.docx]
